# Effect of Dieckol, a Component of *Ecklonia cava*, on the Promotion of Hair Growth

**DOI:** 10.3390/ijms13056407

**Published:** 2012-05-23

**Authors:** Jung-Il Kang, Sang-Cheol Kim, Min-Kyoung Kim, Hye-Jin Boo, You-Jin Jeon, Young-Sang Koh, Eun-Sook Yoo, Sung-Myung Kang, Hee-Kyoung Kang

**Affiliations:** 1 Department of Medicine, School of Medicine, Jeju National University, 102 Jejudaehakno, Jeju 690-756, Korea; E-Mails: asdkji@hanmail.net (J.-I.K.); 25008@hanmail.net (S.-C.K.); loveis6776@hanmail.net (M.-K.K.); wonsein2000@nate.com (H.-J.B.); yskoh7@jejunu.ac.kr (Y.-S.K.); eunsyoo@jejunu.ac.kr (E.-S.Y.); 2 Aqua Green Technology Co. Ltd., 209 Jeju Bio-Industry Center, 102 Jejudaehakno, Jeju 690-121, Korea; E-Mail: youjinj@jejunu.ac.kr; 3 Department of Marine Life Science, Jeju National University, 102 Jejudaehakno, Jeju 690-756, Korea; E-Mail: tjdaud81@hanmail.net

**Keywords:** *Ecklonia cava*, dieckol, hair growth, dermal papilla cells, 5α-reductase activity, vibrissa follicle, C57BL/6 mice

## Abstract

This study was conducted to evaluate the effect of *Ecklonia cava*, a marine alga native to Jeju Island in Korea, on the promotion of hair growth. When vibrissa follicles were cultured in the presence of *E. cava* enzymatic extract (which contains more than 35% of dieckol) for 21 days, *E. cava* enzymatic extract increased hair-fiber length. In addition, after topical application of the 0.5% *E. cava* enzymatic extract onto the back of C57BL/6 mice, anagen progression of the hair-shaft was induced. The treatment with *E. cava* enzymatic extract resulted in the proliferation of immortalized vibrissa dermal papilla cells (DPC). Especially, dieckol, among the isolated compounds from the *E. cava* enzymatic extract, showed activity that increased the proliferation of DPC. When NIH3T3 fibroblasts were treated with the *E. cava* enzymatic extract and the isolated compounds from the *E. cava* enzymatic extract, the *E. cava* enzymatic extract increased the proliferation of NIH3T3 fibroblasts, but the isolated compounds such as eckol, dieckol, phloroglucinol and triphlorethol-A did not affect the proliferation of NIH3T3 fibroblasts. On the other hand, the *E. cava* enzymatic extract and dieckol significantly inhibited 5α-reductase activity. These results suggest that dieckol from *E. cava* can stimulate hair growth by the proliferation of DPC and/or the inhibition of 5α-reductase activity.

## 1. Introduction

Androgenetic alopecia (AGA), the most common type of alopecia, is a problem in men over the age of 40. Hair loss is a growing trend in the world, however, the prevention of this condition is not simple [1]. Numerous possibilities have been discussed to treat hair loss [2]. Nevertheless, only two FDA-approved drugs have been available for AGA patients, *i.e.*, finasteride and minoxidil [3,4]. Finasteride is a type II 5α-reductase inhibitor. It can be used in prostatic hypertrophy [5] as well as in hair loss [6]. Minoxidil was used as a drug to treat high blood pressure [7]. However, it was also found to promote hair growth [8,9], although the mechanism of minoxidil-induced hair growth is not fully understood. In a previous study, minoxidil was shown to have proliferative and anti-apoptotic effects on dermal papilla cells [10]. Moreover, it was also found to stimulate hair growth by the opening of ATP-sensitive K^+^-channel [11,12], the up-regulation of vascular endothelial growth factor (VEGF) [13] and the activation of the β-catenin pathway [14] in dermal papilla cells (DPC). The DPC consist of a cluster of specialized fibroblasts that play important roles in the regulation of the hair cycle through the secretion of diffusible proteins, such as insulin-like growth factor-1 (IGF-1) [15], hepatocyte growth factor (HGF) [16], VEGF [13] and transforming growth factor-β (TGF-β) [17,18].

*Ecklonia cava*, a brown alga, grows in the regions of Jeju Island in Korea. In previous reports, *E. cava* has been found to have effects such as anti-bacterial [19] and anti-inflammatory activity [20]. *E. cava* has been reported to contain various phlorotannins—such as eckol, 8,8′-bieckol, 8,4″-dieckol, phlorofucofuroeckol A, triphlorethol-A, phloroglucinol, dioxinodehydroeckol, fucodiphlorethol G [21–24]. Eckol was reported to have radioprotective activities [25], dieckol to have anti-inflammatory effects [26], and triphlorethol-A to have antioxidant activity [22,23]. Recently, it has been found that phloroglucinol induced apoptosis [24]. However, the effect and the underlying mechanisms of *E. cava* on hair growth have not been described. In this study, we examined the promotion effects and the mechanisms of *E. cava* extract and its components (eckol, dieckol, triphlorethol-A and phloroglucinol) on hair growth.

## 2. Results

### 2.1. The Effect of *Ecklonia cava* Enzymatic Extract on the Hair-Fiber Elongation of Rat Vibrissa Follicle

**Figure 1:**
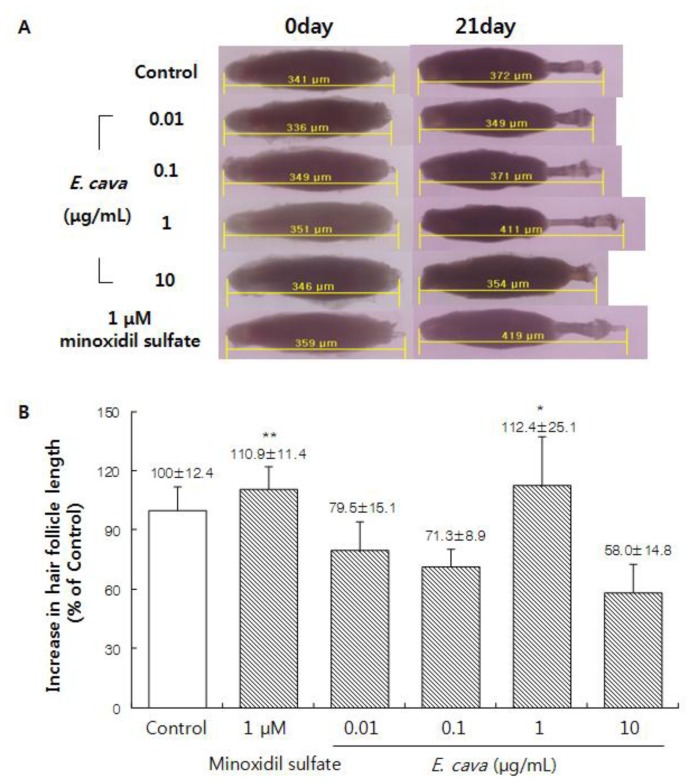
Hair growth effect of *Ecklonia cava* enzymatic extract on rat vibrissa follicles. (**A**,**B**) Individual vibrissa follicles from Wistar rats were micro dissected and then cultured in William’s E medium at 37 °C under 5% CO_2_. Vibrissa follicles were treated with various concentrations of *E. cava* enzymatic extract and minoxidil sulfate, as indicated. All experiments were performed in triplicate. The difference in the length of vibrissa follicles of the control group on day 21 was taken to be 100%. Data are presented as the percentage of the length of the treated follicles based on the mean length of the control follicles ± SE. * p < 0.05, ** p < 0.01 *vs.* control.

To determine whether *E. cava* enzymatic extract could induce hair growth, we examined the effect of *E. cava* enzymatic extract with the use of an organ culture of the rat vibrissa follicle. When the rat vibrissa follicles were treated with various concentrations of *E. cava* enzymatic extract for three weeks, in particular, the hair-fiber length with 1 μg/mL of *E. cava* enzymatic extract treatment showed a significant increase when compared to the control group (Figure 1). The result indicates that *E. cava* enzymatic extract contains components promoting hair growth.

### 2.2. The Effect of *Ecklonia cava* Enzymatic Extract on the Anagen Induction in C57BL/6 Mice

**Figure 2:**
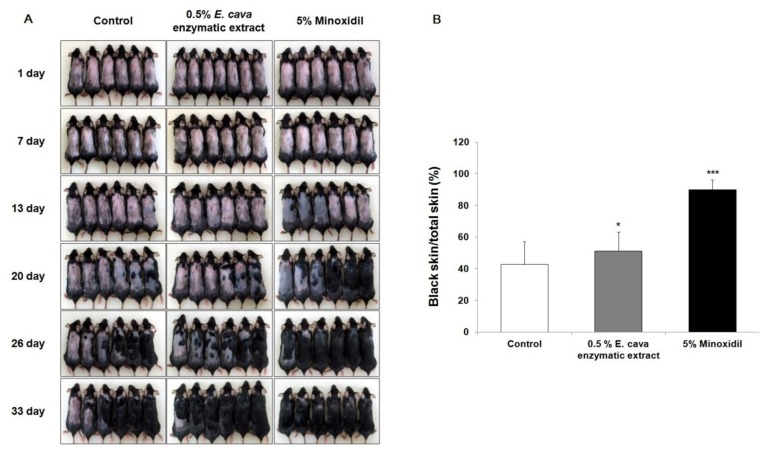
The effect of *Ecklonia cava* enzymatic extract on the anagen induction in C57BL/6 mice. After shaving, the back skins were treated with 0.5% *E. cava* enzymatic extract, vehicle and 5% minoxidil every day for 33 days. (**A**) The back skins were photographed at 1, 7, 13, 20, 26 and 33 days after depilation; (**B**) On day 26, the quantitative assessment of anagen induction analyzed via dotmatrix planimetry was performed. The transparency was put on a photo of a mouse to mark the areas that were in different stages (pink = telogen, anagen = black). Afterward a dotmatrix (sheet with a uniform defined dot pattern) was placed under the marked foil to calculate the percentages of the regions of interest by counting the dots. The percentage of anagen induction was calculated by the equation [(black skin/total skin) × 100]. Data are presented as the mean ± SE (*n* = 6). * p < 0.05, ** p < 0.01, *** p < 0.001 *vs.* vehicle treated control.

To investigate whether anagen induction was promoted by *E. cava* enzymatic extract, we used C57BL/6 mice, since the dorsal hair is known to have a time-synchronized hair growth cycle [27]. Shaved skin of telogen C57BL/6 mice is pink, which then darkens along with anagen initiation. As shown in Figure 2, the area of black skin was significantly larger (*p* < 0.05) in the 0.5% *E. cava* enzymatic extract treated group than in the control group at 26 days after depilation. Induction of the anagen phase in the 0.5% *E. cava* enzymatic extract treated group was observed to be faster than in the control group. The 5% Minoxidil (MINOXYL^TM^) treated group, a positive control group, showed gray skin from 13 days after depilation.

### 2.3. The Effects of *Ecklonia cava* Enzymatic Extract and Its Isolated Compounds on the Proliferation of Dermal Papilla Cells

**Figure 3:**
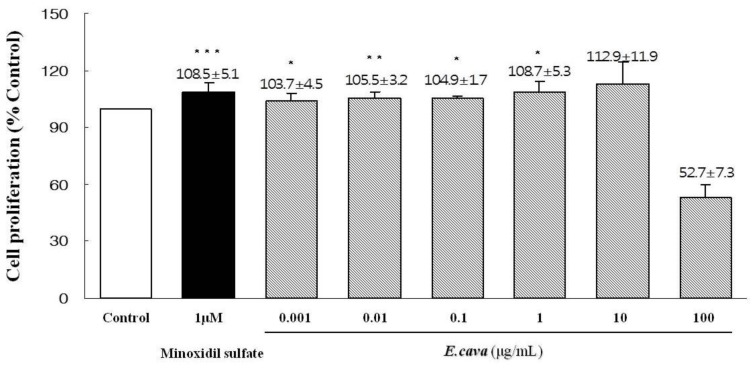
The effect of *Ecklonia cava* enzymatic extract on the proliferation of dermal papilla cells. Rat vibrissa immortalized DPC (1.0 × 10^4^ cells/mL) were plated in 96 well plates. DPC were treated with various concentrations of *E. cava* enzymatic extract and minoxidil sulfate, as indicated. Cell proliferation was measured using a MTT assay for 4 days. All experiments were performed in triplicate. Data are presented as the mean ± SD. * p < 0.05, ** p < 0.01, *** p < 0.001 *vs.* control.

We examined the effects of *E. cava* enzymatic extract and its isolated compounds on the proliferation of DPC. When DPC were treated with *E. cava* enzymatic extract in the concentrations of 0.001, 0.01, 0.1, 1, 10 and 100 μg/mL, *E. cava* enzymatic extract significantly promoted the proliferation of DPC compared with the vehicle-treated control at all the concentrations, except the 100 μg/mL (Figure 3). We evaluated whether the isolated compounds from *E. cava* enzymatic extract such as eckol, dieckol, phloroglucinol and triphlorethol-A, could promote the proliferation of DPC. DPC were treated with eckol, dieckol, phloroglucinol and triphlorethol-A, individually, at 0.005, 0.01, 0.05, 0.1, 0.5, 1 and 10 μg/mL for 4 days. The dieckol increased the proliferation of DPC by 100.5%, 103.9%, 113.5%, 106.1%, 108.1%, 98.5% and 97.3%, respectively (Table 1). The eckol also promoted the proliferation of DPC by 100.8%, 106.1%, 120.3%, 108.5%, 107.8%, 105.4% and 104.1%, respectively (Table 1). However, phloroglucinol and triphlorethol-A did not affect the proliferation of DPC (Table 1).

### 2.4. The Effects of *Ecklonia cava* Enzymatic Extract and Its Isolated Compounds on the Proliferation of NIH3T3 Fibroblasts

**Figure 4:**
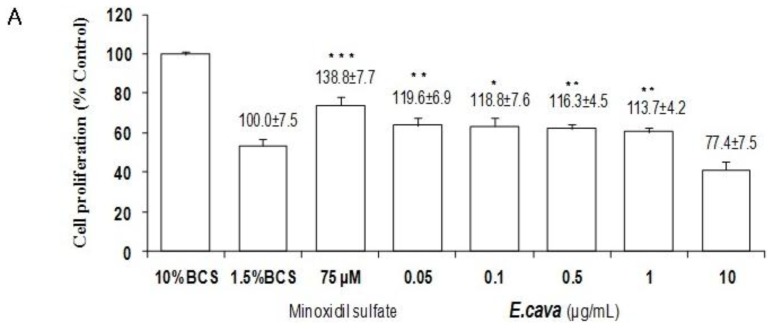

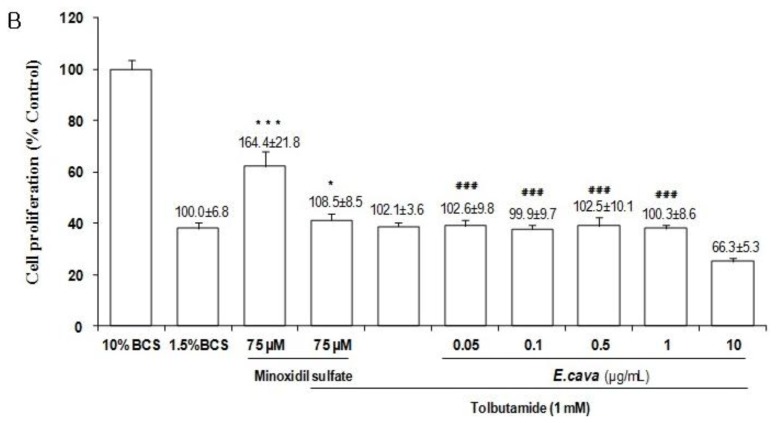
The effect of *Ecklonia cava* enzymatic extract on the proliferation of NIH3T3 fibroblasts. (**A**,**B**) Mouse embryonic NIH3T3 fibroblasts (1.0 × 10^4^ cells/mL) were plated in 96 well plates. NIH3T3 fibroblasts were treated with various concentration of *E. cava* enzymatic extract*,* as indicated. Stimulation with minoxidil served as a positive control. (**B**) NIH3T3 fibroblasts were pretreated with tolbutamide for 30 min prior to incubation with *E. cava* enzymatic extract. Cell proliferation was measured using a MTT assay for four days. All experiments were performed in triplicate. Data are presented as the mean ± SD. * p < 0.05, ** p < 0.01, *** p < 0.001 *vs.* control; ^###^ *p* < 0.001 *vs. E. cava*-treated group.

Minoxidil, a hair-growth promoting agent, has a mitotic effect on NIH3T3 fibroblasts via K_ATP_ channel opening. Whether *E. cava* enzymatic extract, eckol, dieckol, phloroglucinol and triphlorethol-A could act as an opener of K_ATP_ channel, proliferation of NIH3T3 fibroblasts was examined. NIH3T3 fibroblasts were treated with *E. cava* enzymatic extract, eckol, dieckol, phloroglucinol and triphlorethol-A at 0.05, 0.1, 0.5, 1 and 10 μg/mL. The *E. cava* enzymatic extract significantly increased the proliferation of NIH3T3 fibroblasts by 119.6%, 118.8%, 116.3%, 113.7% and 77.4%, respectively (Figure 4A). To evaluate whether the *E. cava*-induced proliferation was mediated through K_ATP_ channel opening, NIH3T3 fibroblasts were pretreated with tolbutamide, a non-selective blocker of K^+^ channels. Tolbutamide inhibited the *E. cava*-induced proliferation of NIH3T3 fibroblasts (Figure 4B). Nevertheless, it is important to note that dieckol, a major component of the *E. cava* enzymatic extract, did not alter the proliferation of NIH3T3 fibroblasts (data not shown). Eckol, phloroglucinol and triphlorethol-A slightly increased the proliferation of NIH3T3 fibroblasts compared with the control group (data not shown).

### 2.5. The Effects of *Ecklonia cava* Enzymatic Extract and Its Isolated Compounds on 5α-Reductase Activities

**Figure 5:**
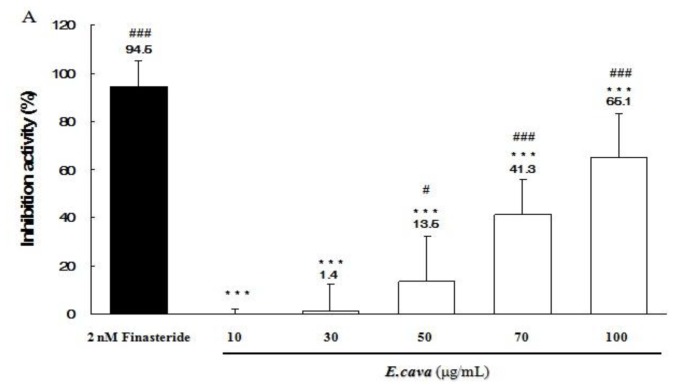

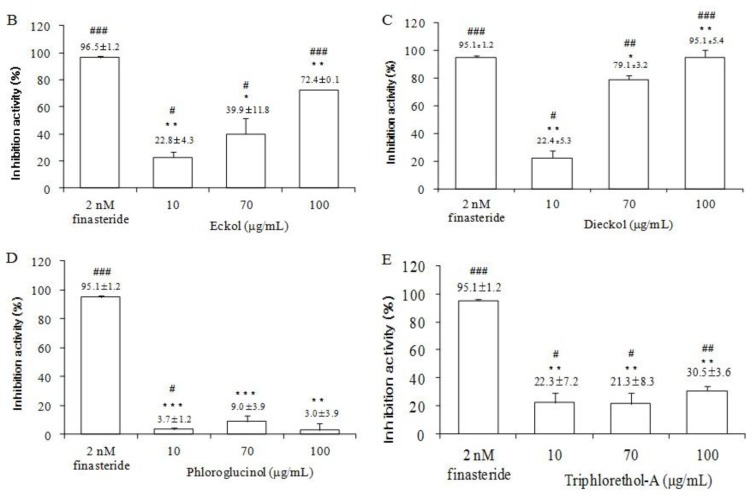
Inhibition effects of *Ecklonia cava* enzymatic extract and its isolated compounds on 5α-reductase activities. (**A**-**E**) Assay of 5α-reductase inhibition was performed using a crude extract of rat prostate. The reaction mixture contained [1,2,6,7-^3^H] testosterone, prostatic enzyme and *E. cava* enzymatic extract or its isolated compounds (eckol, dieckol, phloroglucinol and triphlorethol-A). The conversion rate of testosterone (T) to dihydrotestosterone (DHT) was calculated by the equation [DHT/(T + DHT)]. Inhibition activity (%) was expressed as a percentage of reduced conversion rate compared to the control. The inhibition activity of control group was regarded as 0% (not shown). Finasteride was used as a positive control. Data are presented as the mean ± SD of three independent experiments. * p < 0.05, ** p < 0.01, *** p < 0.001 *vs.* finasteride; ^#^ *p* < 0.05, ^##^ *p* < 0.01, ^###^ *p* < 0.001 *vs.* control.

5α-reductase activity is known to be important for preventing hair loss. We investigated the effects of *E. cava* enzymatic extract, eckol, dieckol, phloroglucinol and triphlorethol-A on the 5α-reductase activity using rat prostatic enzyme. As shown in Figure 5, the *E. cava* enzymatic extract, eckol and dieckol significantly inhibited 5α-reductase activities in a dose-dependent manner (Figure 5A–C). Especially, when the reaction mixture was incubated with 100 μg/mL of dieckol, its inhibition activity was similar to that of the finateride treated group, a positive control group (Figure 5C). However, phloroglucinol did not affect 5α-reductase activities (Figure 5D). 5α-Reductase activities in the triphlorethol-A treated group showed a slight inhibition (Figure 5E).

## 3. Discussion

In this study, the hair growth promoting effect of *E. cava* enzymatic extract, which contains more than 35% of dieckol, was investigated *in vitro* and *in vivo*. To the best of our knowledge, this study is the first to demonstrate that *E. cava* enzymatic extract and dieckol, a component of *E. cava*, have the potential to promote hair growth via the proliferation of dermal papilla cells and/or the inhibition of 5α-reductase activity.

The hair growth cycles, in hair follicle organ cultures of the rat vibrissa follicles, have been reported to be synchronized according to their age [28] and the isolated rat vibrissa follicles could be maintained *in vitro* up to 23 days [29]. Use of the organ culture methods to evaluate hair follicle growth is thought to be correlated with *in vivo* systems because the extent of hair growth can be observed as the sum of the function of each cell [30]. We found that the *E. cava* enzymatic extract increased the hair-fiber length of follicles by 12.4% at 1 μg/mL, compared with the vehicle-treated control. To evaluate the *in vivo* effect of *E. cava* enzymatic extract on the induction of the anagen phase, the hair growth promoting effect on C57BL/6 mouse was examined. The hair growth stimulating *in vitro* effect of *E. cava* enzymatic extract was also observed *in vivo* using C57BL/6 mice.

The hair follicle consists of several distinct epithelial cells and DPC [31,32]. Regulation of hair growth depends on the balance between proliferation and apoptosis in the DPC [10]. As shown in figure 3, *E. cava* enzymatic extract significantly increased the proliferation of DPC. We also examined whether the isolated compounds of *E. cava* extracts such as eckol, dieckol, phloroglucinol and triphlorethol-A, could increase the proliferation of DPC. We observed that eckol and dieckol increased the proliferation of DPC, whereas phloroglucinol and triphlorethol-A did not affect proliferation of DPC (Table 1).

K^+^ channel opening is involved in not only mitogenesis [33], but also hair growth [34]. In previous studies, minoxidil was able to potentiate the mitogenic effects on NIH3T3 fibroblasts through the K_ATP_ channel opening [35]. As shown in Figure 4, the *E. cava* enzymatic extract could promote the proliferation of NIH3T3 fibroblasts, which was inhibited by tobutamide, a non-selective blocker of K^+^ channels. The result indicates that the *E. cava* enzymatic extract can promote hair growth via the K_ATP_ channel opening.

Inhibition of 5α-reductase activity is important in preventing hair loss in AGA [36,37]. *E. cava* enzymatic extract significantly inhibited 5α-reductase activity in a dose dependent manner. Among the isolated compounds from *E. cava* enzymatic extract—eckol, dieckol, phloroglucinol and triphlorethol-A—dieckol was the most active. The results suggest that *E. cava* enzymatic extract and dieckol could have the potential for the treatment of AGA via the inhibition of 5α-reductase activities. Previous studies propose that AGA may be caused by DHT in different ways: The miniaturization of dermal papilla and hair follicles is induced by DHT, which leads to transition from anagen to catagen [38]. DHT increases the levels of transforming growth factor-β1 (TGF-β1) and TGF-β2 in dermal papilla cells, which leads to decreased proliferation of epithelial cells [39,40]. Up-regulation of dickkopf related protein-1 (DKK-1) by DHT can cause repression of the growth of epithelial cells in hair follicles [41]. In further studies, we need to examine whether that *E. cava* enzymatic extract and dieckol can regulate the levels of TGF-β1/β2 and DKK-1 in dermal papilla cells. The androgen action and gene expressions in dermal papilla cells (DPCs) from the human beard are known to be different from those in DPCs of the human scalp. In the future, therefore, although the structure of hair follicle in the human scalp is very similar to that of hair follicle in rat vibrissa, we need to examine whether *E. cava* extract and dieckol can promote hair growth of the human scalp.

## 4. Experimental Section

### 4.1. Alga Material

The brown alga, *E. cava*, was collected along the coasts of Jeju Island in Korea, between February and May 2010 and taxonomically identified by Professor Ki Wan Lee. The samples were washed three times in tap water to remove any attached salt, epiphytes, and sand. Then, they were rinsed carefully with fresh distilled water, and maintained in a medical refrigerator at −20 °C. The frozen samples were then lyophilized and homogenized using a grinder prior to extraction.

### 4.2. Preparation of *E. cava* Enzymatic Extract

We followed the methods reported in previous studies for the preparation of *E. cava* enzymatic extract [42]. To briefly state the preparation procedure, fifty grams of *E. cava* were homogenized with water (2 L), and mixed with 500 μL of carbohydrate enzyme (celluclast 1.5L FG, Novozyme Nordisk, Bagsvaerd, Denmark). *E. cava* enzymatic extract was adjusted to be within the optimum pH and temperature range of the carbohydrate enzyme and enzymatic reactions were performed for 24 h. Following extraction, the extract was boiled for 10 min at 100 °C to inactivate the enzymes. Then, *E. cava* enzymatic extract was clarified by centrifugation (3000 rpm, for 20 min at 4 °C) to remove the residue. *E. cava* enzymatic extract was adjusted to pH 7.0.

### 4.3. Extraction and Isolation of Phlorotannins from *Ecklonia cava*

Eckol, dieckol, phloroglucinol and triphlorethol-A were isolated from *E. cava* as previously described [43]. In short, the dried *E. cava* was extracted three times with 80% aqueous EtOH, and was evaporated in a vacuum. The EtOH extract was then partitioned with EtOAc. The EtOAc fraction was subjected to silica and LH-20 column chromatography. The active compounds were finally purified by reversed-phase HPLC (ThermoFisher Scientific, San Jose, CA, USA), and the purified compounds were then confirmed by comparing their LC/MS, ^1^H NMR data to those in the existing literature [43].

Eckol: LC/MS data (M^+^, *m/z*: 372.0 calcd for C_18_H_12_O_9_). ^1^H NMR (400 MHz, DMSO-*d*6) δ 9.54 (1H, s, OH-9), 9.45 (1H, s, OH-4), 9.21 (2H, s, OH-2,7) 9.16 (2H, s, OH-3′,5′), 6.14 (1H, s, H-3), 5.96 (1H, d, J = 2.8 Hz, H-8), 5.80 (1H, d, J = 1.7 Hz, H-4′), 5.78 (1H, d, J = 2.8 Hz, H-6), 5.72 (2H, J = 1.7 Hz, H-2′,6′).

Dieckol: LC/MS data (M^+^, *m/z*: 742.0 calcd for C_36_H_22_O_18_). ^1^H NMR (400 MHz, DMSO-*d*6) δ 9.71(1H, s, OH-9), 9.61 (1H, s, OH-9″), 9.51 (1H, s, OH-4″), 9.46 (1H, s, OH-4), 9.36 (2H, s, OH-3″,5″), 9.28 (1H, s, OH-2″), 9.23 (1H, s, OH-2), 9.22 (1H, s, OH-7″), 9.15 (2H, s, OH-3′,5′) 6.17 (1H, s, H-3″), 6.14 (1H, s, H-3), 6.02 (1H, d, J = 2.7 Hz, H-8), 5.98 (1H, d, J = 2.7 Hz, H-8″), 5.95 (1H, s, H-2′, 6″′), 5.82 (1H, d, J = 2.7 Hz, H-6), 5.81 (1H, d, J = 2.7 Hz, H-6″), 5.80 (1H, t, J = 2.0 Hz, H-4′), 5.78 (2H, d, J = 2.0 Hz, H-2′,6′).

Phloroglucinol: LC/MS data (M^+^, *m/z*: 126 calcd for C_6_H_6_O_3_). ^1^H NMR (400 MHz, DMSO-*d*6) δ 8.97 (3H, s, OH-1,3,5), 5.66 (3H, s, H-2,4,5).

Triphlorethol-A: LC/MS data (M^+^, *m/z*: 374.0 calcd for C_18_H_14_O_9_). ^1^H NMR (400 MHz, DMSO-*d*6) δ 5.7 (1H, d, *J* = 2.7, H-3), 6.0 (1H, d, *J* = 2.9, H-5), 5.8 (1H, S, H-3′), 5.8 (1H, S, H-5′), 6.0 (1H, d, *J* = 2.2, H-2″), 5.9 (t, *J* = 2.2, H-4″), 6.0 (1H, d, *J* = 2.2, H-6″).

**Figure 6:**
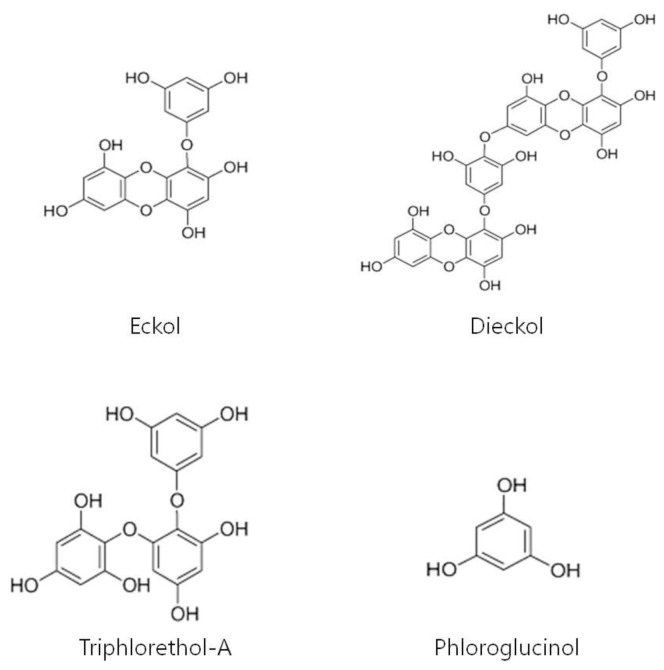
Structures of eckol, dieckol, phloroglucinol and triphlorethol-A.

The purity of eckol, dieckol, phloroglucinol and triphlorethol-A was >95%, according to the peak area of all components absorbed at each specific wavelength in HPLC analysis. Their chemical structures are shown in Figure 6, and were freshly dissolved in dimethyl sulfoxide (DMSO) (Sigma, St. Louis, MO, USA) for subsequent treatment. Further, minoxidil sulfate and minoxidil were also dissolved in DMSO for subsequent treatment. The final concentration of DMSO was adjusted to 0.2% (v/v) in the following experiment. Tolbutamide was made up as a 410 mM stock solution in ethanol and added to the culture media in a final concentration of 0.25% ethanol.

### 4.4. Animals

Male Wistar rats (3 weeks of age) were supplied from Orient Bio (Seongnam, Gyeonggi, Korea). Six-week-old female C57BL/6 mice and 8-week-old male spargue-Dawley (SD) rats were purchased from Dae-Han Biolink (Eumsung, Chungbuk, Korea) and were provided with a standard laboratory diet and water *ad libitum*. All animals were cared for by using protocols (20100031) approved by the Institutional Animal Care and Use Committee (IACUC) of the Jeju National University.

### 4.5. Isolation and Culture of Rat Vibrissa Follicles

Isolation of rat vibrissa follicles was performed as described previously [29]. Briefly, rat vibrissa follicles were harvested from male Wistar rats that were 23 days old. To accomplish this, the rats were sacrificed under carbon dioxide (CO_2_). Next, both the left and right mystacial pads were removed from the rats and placed in a 1:1 (v/v) solution between Earle’s balanced salts solution (EBSS, Sigma, St. Louis, MO, USA) and PBS that contained 100 unit/mL of penicillin and 100 μg/mL of streptomycin. Anagen vibrissa follicles were then carefully dissected under a stereomicroscope (Olympus, Tokyo, Japan) from posterior parts of the mystacial pads, with considerable caution to remove the surrounding connective tissue without damaging the vibrissa follicle. Using this method, we were able to routinely isolate more than 40 follicles from each animal. The isolated follicles were then placed in separate wells in 24-well plates that contained 500 μL of Williams medium E (GIBCO Inc, Grand Island, NY, USA) supplemented with 2 mM L-glutamine (Gibco Inc, Grand Island, NY, USA), 10 μg/mL insulin (Sigma, St. Louis, MO, USA), 50 nM hydrocortisone (Sigma, St. Louis, MO, USA), 100 unit/mL penicillin and 100 μg/mL streptomycin at 37 °C. They were cultivated in an atmosphere comprised of 5% CO_2_ and 95% air. The isolated follicles were then treated with vehicle (DMSO diluted 1:1000 in Williams medium E) as a control and *E. cava* enzymatic extract (0.01, 0.1, 1 and 10 μg/mL). Minoxidil sulfate (Sigma, St. Louis, MO, USA) was used as a positive control in the culture systems (Buhl *et al.*, 1990). The culture medium was changed every 3 days and photographs of the cultured vibrissa follicles were taken using a stereomicroscope, for 3 weeks. The length of the hair follicles was measured using a DP controller (Olympus, Tokyo, Japan).

### 4.6. Hair Growth Activity *in Vivo*

Anagen was induced on the back skin of C57BL/6 mice that were in the telogen phase of the cycle by depilation, as described previously [27]. Briefly, 6-week-old female C57BL/6 mice were allowed to adapt to their new environment for one week. The anagen was then induced in the back skin of the 7-week-old female C57BL/6 mice by shaving, which led to synchronized development of anagen hair follicles. From the following day (day 1), 0.2 mL of 0.5% *E. cava* enzymatic extract in 50% ethanol was topically applied every day for 33 days. 5% Minoxidil (MINOXYL^TM^; Hyundai Pharm. Co. Ltd., Cheonan, Chungnam, Korea) was used as a positive control. The back skin of the mice was then observed and photographed at 1, 7, 13, 20, 26 and 33 days after shaving. For the quantitative assessment, dotmatrix planimetry was performed [44].

### 4.7. Culture and Proliferation Assay of Dermal Papilla Cells

Rat vibrissa immortalized dermal papilla cell line [45] was donated by the Skin Research Institute, Amore Pacific Corporation R & D Center, South Korea. The DPC were cultured in DMEM (Hyclone Inc., Logan, UT, USA), supplemented with 10% fetal bovine serum (Gibco BRL, Grand Island, NY, USA) and penicillin/streptomycin (100 unit/mL and 100 μg/mL, respectively), at 37 °C in a humidified atmosphere under 5% CO_2_.

The proliferation of DPC was evaluated by measuring the metabolic activity using a 3-[4,5-dimethylthiazol-2-yl]-2,5-diphenyltetrazolium bromide (MTT) [46]. Briefly, DPC at 1.0×10^4^ cells/mL were seeded into 96-well plate, then cultured for 24 h in a serum-free DMEM, and then treated with vehicle (DMSO diluted 1:1000 in serum-free DMEM) as a control, *E. cava* extract (0.001~100 μg/mL), eckol (0.005~10 μg/mL), dieckol (0.005~10 μg/mL), phloroglucinol (0.005~10 μg/mL), triphlorethol-A (0.005~10 μg/mL) and minoxidil sulfate (1 μM), for 4 days. After incubation, 0.1 mg (50 μL of a 2 mg/mL solution) of MTT (Sigma, St. Louis, MO, USA) was added to each well, and the cells were then incubated at 37 °C for 4 h. Next, the plates were centrifuged at 1000 rpm for 5 min at room temperature and the media was then carefully aspirated. 200 μL of DMSO was then added to each well to dissolve the formazan crystals and the absorbance of the plates, at 540 nm, was then read immediately on a microplate reader (BioTek Instrument, Inc., Winooski, VT, USA). All experiments were performed three times and the mean absorbance values were calculated. The results are expressed as a percentage of absorbance caused by treatment with the extract or the active component compared to those of the vehicle treated controls.

### 4.8. Culture and Proliferation Assay of NIH3T3 Fibroblasts

The mouse embryonic NIH3T3 fibroblasts were purchased from ATCC (Rockville, MD, USA) and cultured in ATCC-formulated Dulbecco’s Modified Eagle’s Medium (DMEM), supplemented with 10% (v/v) heat-activated bovine calf serum (BCS), 100 unit/mL penicillin and 100 μg/mL streptomycin at 37 °C atmosphere and 5% CO_2_.

The proliferation of NIH3T3 fibroblasts was also evaluated by measuring the metabolic activity using MTT assay [46]. NIH3T3 fibroblasts, at 1.0 × 10^4^ cells/mL, were seeded into a 96-well plate. Cells were incubated for 24 h with DMEM supplemented with 10% BCS, then washed with phosphate buffered saline (PBS, Sigma, St. Louis, MO, USA). The cells were maintained with DMEM supplemented with 10% BCS or 1.5% BCS and treated with vehicle (DMSO) as a control, *E. cava* extract (0.05~10 μg/mL), eckol (0.05~10 μg/mL), dieckol (0.05~10 μg/mL), phloroglucinol (0.05~10 μg/mL), triphlorethol-A (0.05~10 μg/mL) and minoxidil (75 μM), for 4 days. To clarify whether proliferation of NIH3T3 fibroblasts is regulated by K_ATP_ channel opening, NIH3T3 fibroblasts were pretreated with tolbutamide (2 mM), a non-selective blocker of K^+^ channel, for 30 min prior to incubation with *E. cava* enzymatic extract for 4 days. All experiments were performed three times and the mean absorbance values were calculated. The results are expressed as the percentage in the absorbance caused by treatment with the extract or the active component compared to those of the vehicle untreated controls.

### 4.9. Assay of Rat Prostatic 5α-Reductase

Male SD rats (8 weeks) were sacrificed with carbon dioxide (CO_2_). The prostates of rats were dissected, freed of their capsules, then washed with saline, and stored at −80 °C. Frozen tissues were thawed on ice and procedures were carried out at 4 °C. The tissues were homogenized with Polytron homogenizer (Brinkman Instruments, Wesrbury, NY, USA) in 5–6 tissue volumes of medium A (0.32 M sucrose, 1 mM dithiothreitol (DTT), 0.2 mM phenylmethylsulfonylfluoride (PMSF), and 20 mM potassium phosphate buffer pH 6.6). The homogenates were centrifuged at 100,000 g for 60 min. The pellets were recovered, washed with three tissue volumes of medium A and centrifuged two additional times at 400 g at 0 °C for 10 min. The washed pellets were suspended in medium A and stored at −80 °C until use. The suspension (2.5 mg protein/mL for Rat prostates, determined by the Bradford method) was used as source of 5α-reductase.

5α-reductase activities were assayed as previously described [47]. The reaction mixture contained a final volume of 500 μL: one millimole DTT, 40 mM potassium phosphate buffers, 2 mM NADPH, Testosterone including 120 n Ci [1,2,6,7-^3^H]. The reaction in triplicate was started when it was added to the rat prostatic enzyme fraction (250 μg protein), 0.2% DMSO as a control, *E. cava* extract (10, 30, 50, 70 and 100 μg/mL), eckol (10, 70 and 100 μg/mL), dieckol (10, 70 and 100 μg/mL), phloroglucinol (10, 70 and 100 μg/mL) and triphlorethol-A (10, 70 and 100 μg/mL). Finasteride 2 nM (MERCK SHARP & DOHME, South Granville, Australia) was used as a positive control. The mixture was incubated at 37 °C for 60 min, and then stopped by adding 1 mL of ethyl acetate and mixing for 1 min. After centrifugation at 1000 g for 5 min, the organic phase was removed which then was dried under a heating plate, dissolved in 50 μL of ethyl acetate containing 500 μg/mL testosterone and 500 μg/mL dihydrotestosterone (DHT) and applied to a silica gel 60 F254 TLC plate (Merck, Darmstadt, Germany). The plate was developed in a solvent system consisting of an ethyl acetate:cyclohexane (1:1) solution, the plate then was air dried. Testosterone was visibly seen under the UV light (254 nm) and DHT was detected using 10% H_2_SO_4_ solution via posteriorly heating the plate. Under these conditions, DHT will be shown as a dark yellow color. Areas containing androgen were removed and the strips were soaked in the 5 mL of ULTIMA GOLD^TM^ Cocktails (PerkinElmer, Inc., Waltham, MA, USA) and the radioactivity level was then measured via a liquid scintillation counter (Packard Bioscience, Meriden, CT, USA). The activity of 5α-reductase was expressed as the ratio [DHT/(T + DHT)] × 100.

### 4.10. Statistical Analyses

Each experiment was performed at least in triplicate. Results are expressed as mean ± SD or mean ± SE from three separate experiments. The Student’s *t* test and one-way ANOVA test were used to determine the statistical significance.

## 5. Conclusions

In conclusion, this study demonstrated that dieckol, a principal component of *E. cava*, could stimulate hair growth through the proliferation of dermal papilla cells and the inhibition of 5α-reductase activity. These finding indicate that dieckol from *E. cava* enzymatic extract is a possible therapeutic compound for treatment of hair loss.

## Figures and Tables

**Table 1:** The effects of isolated compounds from *Ecklonia cava* enzymatic extract on the proliferation of dermal papilla cells.

Concentration (μg/mL)	Compounds			

	Eckol	Dieckol	Phloroglucinol	Triphlorethol-A
0.005	100.8 ± 1.6 *	100.5 ± 4.7 **	102.0 ± 8.5	101.7 ± 7.9
0.01	106.1 ± 4.5 *	103.9 ± 6.5	100.4 ± 6.8	102.9 ± 3.5
0.05	120.3 ± 9.9	113.5 ± 6.2 *	96.4 ± 5.3	100.4 ± 8.2
0.1	108.5 ± 7.7	106.1 ± 5.4	99.6 ± 4.7	99.3 ± 5.2
0.5	107.8 ± 6.7	108.1 ± 6.2	99.2 ± 4.0	99.4 ± 7.7
1	105.4 ± 6.2	98.5 ± 6.5	98.3 ± 2.3	103.3 ± 7.5
10	104.1 ± 9.7	97.8 ± 3.9	76.8 ± 5.6	100.6 ± 4.5
1 μM Minoxidil sulfate		119.0 ± 8.1 ***		

The proliferation of the control group was regarded as 100% (not shown). All experiments were performed in triplicate. Data are presented as the mean ± SD.

* *p* < 0.05,

** *p* < 0.01,

*** *p* < 0.001 *vs.* control.
